# Cytokine changes in patients with heatstroke during pilgrimage to Makkah

**DOI:** 10.1080/09629359791839

**Published:** 1997-04

**Authors:** I. A. Hashim, A. Al-Zeer, S. Al-Shohaib, M. Al-Ahwal, A. Shenkin

**Affiliations:** 1 Department of Pathology King Khalid National Guard Hospital P.O. Box 9515 Jeddah 21423 Saudi Arabia; 2 Department of Medicine King Khalid National Guard Hospital P.O. Box 9515 Jeddah 21423 Saudi Arabia; 3 Department of Medicine King Abdelaziz University Hospital Jeddah Saudi Arabia; 4 Intensive Care Unit King Khalid University Hospital Riyadh Saudi Arabia; 5 Department of Clinical Chemistry Royal Liverpool University Hospital Liverpool UK

## Abstract

Circulating levels and role of IL-6, IL-1ra, TNFsr-II and CRP in patients with heatstroke is not fully known. This study correlated levels of these mediators with outcome in 26 patients. In survivors (*n*=20), IL-6 concentration declined on cooling, whereas in non-survivors levels continued to increase at 6 h following admission before declining. Admission TNFsr-II concentrations in survivors were significantly lower than non-survivors and levels continued to rise in both groups. IL-1ra levels were markedly elevated in both groups. Changes in cytokine levels were not influenced by renal function. Elevated C-reactive protein levels were observed for both groups and remained so despite cooling, furthermore, there was no correlation with alanine aminotransferase levels. The study demonstrated the elevation of the above mediators and suggested a role in the pathogenesis of heatstroke. Markedly elevated levels or those that remained elevated despite cooling were associated with mortality.


Mediators of Inflammation, **6**, 135–139 (1997)

### Cytokine changes in patients with heatstroke during pilgrimage to Makkah

I. A. Hashim,[1,CA] A. Al-Zeer,[4] S. Al-Shohaib,[2] M. Al-Ahwal[3] and A. Shenkin[5]

Departments of [1]Pathology and [2]Medicine, King Khalid National Guard Hospital, P.O. Box 9515, Jeddah 21423; [3]Department of Medicine, King Abdelaziz University Hospital, Jeddah; [4]Intensive Care Unit, King Khalid University Hospital, Riyadh, Kingdom of Saudi Arabia; and [5]Department of Clinical Chemistry, Royal Liverpool University Hospital, Liverpool, UK

[CA]Corresponding Author
Fax: (+966) 2 665 3031

**CIRCULATING levels and role of IL-6, IL-1ra, TNFsr-II and CRP in patients with heatstroke is not fully known. This study correlated levels of these mediators with outcome in 26 patients. In survivors ($n = 20$), IL-6 concentration declined on cooling, whereas in non-survivors levels continued to increase at 6 h following admission before declining. Admission TNFsr-II concentrations in survivors were significantly lower than non-survivors and levels continued to rise in both groups. IL-1ra levels were markedly elevated in both groups. Changes in cytokine levels were not influenced by renal function. Elevated C-reactive protein levels were observed for both groups and remained so despite cooling, furthermore, there was no correlation with alanine aminotransferase levels. The study demonstrated the elevation of the above mediators and suggested a role in the pathogenesis of heatstroke. Markedly elevated levels or those that remained elevated despite cooling were associated with mortality.**

**Key words:** Acute phase reaction, C-reactive protein, Cytokines, Hyperthermia, Interleukin-6

### Introduction

Heatstroke, a true medical emergency, occurs in two distinct settings exertional hyperthermia during hot humid weather, and that due to impaired heat dissipation, mostly seen in sedentary and elderly persons.[1]

Patients with heatstroke have complete loss of thermoregulation, present with core body temperature $> 40°C$, hot dry skin, neurological dysfunction with impaired mental status and occasionally coma.[2] Mortality rate is high where terminal events include shock, arrhythmias, myocardial infarction, renal failure and various neurological dysfunctions.[3]

Cytokines play a major role, both in heat production and in modulating the response to stress and trauma. Interleukin-6 (IL-6), a pleiotropic cytokine, modulates the acute phase response and stimulates hepatic synthesis and release of C-reactive protein (CRP).[4] Other mediators of acute phase response include: tumour necrosis factor alpha (TNFα) and interleukin-1 (IL-1), both modulate the release and activity of IL-6.[5] The activities of TNFα and IL-1 are attenuated by circulating inhibitors namely TNF soluble receptors (TNFsr) I and II[6,7] and IL-1 receptor antagonist (IL-1ra) respectively.[8] These inhibitors may afford a protective mechanism against a continued inflammatory process.

Elevated TNFsr-II levels have been observed in various pathological conditions including endotoxaemia and infection[9,10] and several reports showed correlation between concentrations of TNF and its soluble receptor.[11]

IL-1ra is released under various conditions such as trauma and inflammation.[12,13]

Hepatocytes play a major role in acute phase response. The effect of the heatstroke on hepatocytes and thus on the synthesis and release of CRP and cytokines was assessed by measuring circulating alanine aminotransferase (ALT) levels. Furthermore, the effect of possible renal dysfunction in heatstroke on the circulating levels of these cytokines was assessed by correlating with serum creatinine levels.

The circulating levels and role of these protein mediators in hyperthermia and in the pathogenesis and outcome of heatstroke is unknown. In this study, we measured serum concentrations of stimulators/mediators of the acute phase response (IL-6 and CRP) and inhibitors/neutralizers of cytokine activities

© 1997 Rapid Science Publishers

(TNFsr-II, and IL-1ra) in patients with heatstroke during pilgrimage to Makkah and correlated the circulating levels with outcome.

### Materials and Methods

IL-6, IL-1ra, and TNFsr-II enzyme-linked immunosorbent assay (ELISA) kits were obtained from R&D Systems Europe, Abingdon, Oxfordshire, UK. Analysis was performed according to the supplier's instructions. CRP, creatinine, and ALT assay kits were obtained from Boehringer Mannheim (BM), Hertfordshire, UK, and analysis performed on a Hitachi 911 analyser (BM) according to the supplier's instructions.

### Subjects

Blood samples ($n = 86$) were obtained from 26 patients (eight males) admitted to the heatstroke centre during the 1994 pilgrimage to Makkah. Patients' ages ranged from 30 to 75 years (median 60 years). Patients were classified as suffering from heatstroke if presented with: core body temperature >40.5°C, hot dry skin and impaired mental status. Ethical committee approval was obtained and blood samples were collected at 0, 6, 12 and 24 h following admission. Serum was collected by centrifugation and analysed for creatinine and ALT levels. Aliquots were stored at −70°C until transport on dry ice to Liverpool, UK, where cytokines and CRP measurements were performed.

### Results

Patients' core body temperatures ranged from 40°C to 43.2°C (median 42.3°C). Patients were cooled to 38°C using conventional cooling bed (lukewarm water spray and cool moving air). Cooling took 45 to 180 min (median 120 min). Six patients (three males, three females) died giving a mortality rate of 23%.

Biochemical parameters measured were not normally distributed (Royston's development of the Shapiro-Francia *W* test).[14] A non-parametric Wilcoxon rank sum test was therefore used to determine statistical significance and results are expressed as median and range (Tables 1 and 2).

### IL-6

IL-6 was detected in all samples with values ranging from 4 to 1594 pg/ml (median 195). IL-6 levels at admission were markedly elevated in all patients. There was no significant difference in median IL-6 admission values between survivors and non-survivors (Table 1). IL-6 levels declined on cooling (0.8 pg/ml/min) over the first 6 h in survivors. By contrast, in non-survivors serum concentrations of IL-6 continued to increase 6 h following admission before declining (Table 1).

There was no correlation between cooling time and IL-6 levels at admission ($r^2 = 0.03$). Additionally, there was no correlation between IL-6 levels at 0 and 6 h following admission and core body temperature ($r^2 = -0.03$ and 0.2 respectively).

**Table 1**. Showing median and range values for serum IL-6, TNFsr-II, IL-1ra and CRP concentrations in patients with heatstroke at admission, 6, 12 and 24 h following admission. S: survivors (n = 20); NS: non-survivors (n = 6)

| | | Time (h) | | | |
|---|---|---|---|---|---|
| | | 0 | 6 | 12 | 24 |
| IL-6 (pg/ml) | S | 479 | 113 | 51 | 63 |
| | | 32–1177 | 6–1338 | 4–775 | 5–512 |
| | NS | 890 | 1411 | 608 | 436 (n = 2) |
| | | 89–1326 | 271–1594 | 282–934 | 129–743 |
| | | $p = 0.14$ | $p < 0.01$ | $p < 0.06$ | – |
| TFNsr-II (pg/ml) | S | 307 | 393 | 429 | 522 |
| | | 175–547 | 221–711 | 241–904 | 360–1119 |
| | NS | 526 | 536 | 725 (n = 2) | 1040 (n = 1) |
| | | 347–671 | 368–722 | 638–812 | – |
| | | $p < 0.005$ | $p = 0.19$ | – | – |
| IL-1ra (pg/ml) | S | > 1888 | > 7573 | > 7573 | > 3117 |
| | | 215–> 7573 | 1027–> 7573 | 810–> 7573 | 722–> 7573 |
| | NS | > 5085 | > 7573 | > 7573 | > 7573 (n = 1) |
| | | 200–> 7573 | > 7573–> 7573 | > 7573–> 7573 | – |
| CRP (ml/l) | S | 4.9 | 6.3 | 11.2 | 20.1 |
| | | < 1–43.5 | < 1–42.7 | < 1–64.4 | 10.7–138.8 |
| | NS | 19.3 | 8.6 | 14.9 | 26.5 |
| | | < 1–65.1 | 1–55.1 | 1.7–28.0 (n = 2) | 35.8 (n = 1) |
| | | $p = 0.31$ | $p = 0.74$ | – | – |

**Table 2**. Showing median and range values for serum creatinine and ALT levels in patients with heatstroke at admission, 6, 12 and 24 h following admission. S: survivors (n = 20); NS: non-survivors (n = 6)

| | | Time (h) | | | |
|---|---|---|---|---|---|
| | | 0 | 6 | 12 | 2 |
| Creatinine | S | 142 | 139 | 100 | 102 |
| (μmol/l) | | 58–265 | 62–285 | 37–332 | 50–384 |
| | NS | 167 | 234 | $n = 2$ | 328 (n = 1) |
| | | 63–226 | 146–307 | 169–215 | – |
| | | $p = 0.6$ | $p < 0.002$ | – | – |
| ALT (IU/l) | S | 14 | 26 | 30 | 41 |
| | | 0–235 | 6–469 | 4–372 | 11–253 |
| | NS | 23 | 53 | $n = 2$ | 136 (n = 1) |
| | | 11–63 | 34–114 | 35–118 | – |
| | | $p = 0.1$ | $p < 0.002$ | – | – |

In survivors, there was no correlation between IL-6 and creatinine levels at admission ($r^2 = 0.16$), 6 h ($r^2 = 0.4$), and 12 h ($r^2 = 0.45$), whereas the correlation at 24 h was poor ($r^2 = 0.64$). However, in non-survivors no correlation was observed between IL-6 and creatinine at admission ($r^2 = 0.31$) whereas the correlation at 6 h was poor ($r^2 = 0.56$).

### TNFsr-II

TNFsr-II was detected in all samples. On admission, non-survivors showed significantly ($p < 0.005$) higher concentrations than survivors (Table 1). Furthermore, despite cooling, concentrations in both groups continued to rise following admission. The degree of rise was, however, greater in non-survivors compared with survivors (Table 1).

In survivors, there was no correlation between TNFsr-II and creatinine levels at admission ($r^2 = 0.0$) and 6 h ($r^2 = 0.44$), whereas, the correlation was positive at 12 and 24 h following admission ($r^2 = 0.69$, and 0.76 respectively). In non-survivors, there was no correlation between TNFsr-II and creatinine levels at admission or 6 h following admission ($r^2 = 0.22$, and 0.3 respectively).

### IL-1ra

Markedly elevated IL-1ra concentrations were detected in all samples. The serum concentrations increased markedly following admission in both groups reaching levels above the upper limit of the assay (7573 ng/ml). Among survivors, the serum concentration in some patients fell between 12 and 24 h following admission, but remained markedly elevated (Table 1).

### CRP

Circulating CRP concentrations ranged between non-detectable (< 1 ml/l) and 138.8 mg/l (median 8.7). Levels continued to rise in both survivors and non-survivors, however, there was no significant difference between both groups at all times (Table 1). There was no correlation between peak CRP levels and admission IL-6 concentrations ($r^2 = 0.02$).

There was no correlation between ALT and CRP levels at admission ($r^2 = 0.2$), however, poor positive correlation was observed 6 h following admission ($r^2 = 0.52$).

### Creatinine

Serum creatinine levels ranged from 37 to 384 μmol/l (median 115) in survivors, whereas in non-survivors the levels ranged from 63 to 328 μmol/l (median 189). Median creatinine levels at all times in both groups were above the upper limit of normal (97 μmol/l) (Table 2).

There was no significant difference ($p > 0.2$) between creatinine levels in survivors at all times, and between 0 and 6 h in non-survivors. How-ever, significant difference was found between survivors and non-survivors 6 h following admission with higher levels in the latter (Table 2).

### ALT

Circulating ALT levels ranged from non-detectable to 469 IU/l (median 26) in survivors and between 11 and 136 (median 45.5) in non-survivors. Seventy-five per cent of all patients had ALT level below the upper limit of normal (43 IU/l).

In survivors, levels increased following admission with significant difference between 0 and

6 h, 0 and 12 h, 0 and 24 h following admission ($p < 0.002$), but no significant difference between 6 and 12 h and between 12 and 24 h ($p = 0.5$). In non-survivors no significant difference was found at 0 and 6 h following admission. However, the difference between both groups was significant only at 6 h following admission (Table 2).

### Discussion

This study examined the changes in serum of patients with heatstroke of the acute phase reactant 'C-reactive protein', the major mediator cytokine IL-6, and that of the inhibitors/modulators of the inflammatory response namely IL-1ra and TNFsr-II.

IL-6, IL-1ra, TNFsr-II were detected in all samples, the general pattern being of markedly elevated levels at admission which either declined or continued to increase following cooling.

Although survivors had elevated IL-6 levels, concentrations greater than 900 pg/ml at admission or those which continued to rise were associated with mortality. This finding is analogous to that observed in septic patients where levels greater than 950 pg/ml were associated with increased mortality.[15] The presence of high IL-6 concentrations in heatstroke patients have previously been reported.[16] The findings indicate a possible role for IL-6 in the pathogenesis of heatstroke and of uncontrolled body temperature elevations. Although IL-6 has been suggested to play a role in fever production,[17] we found no correlation between IL-6 concentrations at admission and core body temperature or cooling time. However, cooling was associated with declining IL-6 levels. This finding suggests a possible role of other factors or cytokines, either working independently or in association with IL-6.

In our patients, IL-1ra and TNFsr-II were markedly elevated at admission and whereas IL-1ra in some patients, finally, declined on cooling, TNFsr-II continued to increase throughout the study period.

IL-1ra inhibits IL-1 activity and thus limits the inflammatory response mediated by IL-1.[12] Markedly elevated levels were observed in all patients (Table 1). However, in most samples, levels were above the upper limit of the assay and unfortunately, sample shortage did not allow for repeat measurement and thus limited statistical analysis of data. The markedly elevated IL-1ra may reflect continued IL-1 release or deranged IL-1ra production in excess of requirement.

In this study, the pattern of circulating TNFsr-II was in contrast to that seen for IL-6, where survivors showed markedly elevated levels compared with non-survivors. Furthermore, the levels continued to rise despite cooling in both groups. The degree of rise was greater in non-survivors compared with survivors.

TNFsr-II inhibits TNFα activity and thus modulates its inflammatory action. TNFα levels has been shown[18] to be elevated in heatstroke patients whereas levels decreased following cooling and there was no correlation between body temperature and TNFα. In our study, markedly elevated TNFsr-II levels were observed in both survivors and non-survivors, however, significantly higher levels were associated with mortality (Table 1).

CRP levels increased in all patients and continued to rise despite cooling indicating sustained release by hepatocytes. There was no significant difference between median CRP levels in both survivors and non-survivors. The lack of correlation between admission IL-6 and peak CRP levels suggests a possible role for other mediators.

The possibility that cytokine levels were influenced by renal function, known to be compromised in these patients,[3] was examined. Cytokine levels were correlated with serum creatinine as a reflection of renal function. In non-survivors there was no or poor correlation between creatinine and IL-6 and TNFsr-II levels. Furthermore, in survivors there was no correlation between IL-6, IL-1ra or TNFsr-II and creatinine levels at 0 and 6 h following admission and poor positive correlation at later times. Since the significant differences in cytokine levels between survivors and non-survivors were only observed early at 0 and 6 h following admission, the lack of correlation with creatinine at these times indicates that such differences were not influenced by renal function.

There was no correlation between CRP and ALT levels at admission ($r^2 = 0.2$), however the correlation at 6 h was poor ($r^2 = 0.52$) but significant. Possible hepatic dysfunction suggested by elevated ALT levels at 6 h following admission did not significantly influence CRP levels. However, skeletal muscle also contain significant amount of ALT and it is difficult to ascertain the source of the enzyme, furthermore, sample shortage did not allow for isoenzyme studies.

The mortality rate in our study was higher than previously reported.[3] This may be due to the small sample size or to our patients' settings.

It is known that the ability to eliminate heat is limited among other things by protective clothing, that prevents sweating and evaporative heat loss. Our patients may have exhibited both heatstroke settings; exertional hyperthermia during hot humid weather and impaired heat dissipation. The majority (69%) of our patients were females, and this may be a consequence of dark-coloured, heavier and all-covered-up clothing compared with white-coloured, lighter, relatively exposed clothing in males. Physiological and psychological adaptation to a hot environment can improve heat tolerance,[19] however this was not assessed in this study.

In conclusion, it is clear that circulating levels of IL-6, TNFsr-II, IL-1ra and CRP are significantly elevated in sera from patients with heatstroke. Cytokine concentrations that were markedly elevated at admission or continued to increased despite cooling were associated with poor outcome. Significant changes in cytokine levels between survivors and non-survivors were not influenced by renal or hepatic dysfunction. Further studies are required in a larger group of patients to examine the role of these mediators in the pathogenesis of heatstroke and the effects of different treatments on the circulating cytokine levels and outcome.

### References

1. Knochel JP. Heat stroke and related heat stress disorders. *Dis Mon* 1989; **35**: 301–377.
2. Clowes GHA, O'Donnell TF. Heat stroke. *New Engl J Med* 1974; **291**: 564–567.
3. Keatinge WR, Coleshaw SRK, Clotter F, Mattock MB, Chelliah R. Increased platelet and red cell counts, blood viscosity, and plasma cholesterol levels during heat stress, and mortality from coronary and cerebral thrombosis. *Am J Med* 1986; **81**: 795–800.
4. Hirano T, Akira S, Taga T, Kishimoo T. Biological and clinical aspects of interleukin-6. *Immunology Today* 1190; **12**: 443–449.
5. Heinrich PC, Castell JV, Andus T. Interleukin-6 and the acute phase response. *Biochem J* 1990; **265**: 621–636.
6. Seckinger P, Isaaz S, Dayer JM. Purification and biologic characterization of a specific tumor necrosis factor alpha inhibitor. *J Biol Chem* 1991; **264**: 11966–11973.
7. Chouaib S, Branellec D, Buurman WA. More insight into the complex biology of TNF. *Immunol Today* 1991; **12**: 141–142.
8. Hannum CH, Wilcox CJ, Arend WP, *et al.* Interleukin-1 receptor antagonist activity of a human interleukin-1 inhibitor. *Nature* 1990; **343**: 336–340.
9. VanZeek K, Kohno T, Fischer E, Rock CS, Moldawer LL, Lowry SF. Tumor necrosis factor soluble receptors circulate during experimental and clinical inflammation and can protect against massive tumor necrosis factor alpha in vitro and in vivo. *Proc Natl Acad Sci (USA)* 1992; **89**: 4845–4849.
10. Andus T, Gross V, Holstege A, *et al.* High concentrations of soluble tumor necrosis factor receptors in ascites. *Hematology* 1992; **16**: 749–755.
11. Spinas GA, Keller U, Brockhaus M. Release of soluble receptors for tumor necrosis factor (TNF) in relation to circulating TNF during experimental endotoxinemia. *J Clin Invest* 1992; **90**: 553–556.
12. Arend WP. Interleukin 1 receptor antagonist; a new member of the interleukin 1 family. *J Clin Invest* 1991; **88**: 1445–1451.
13. Fischer E, Marrano MA, VanZee KJ, *et al.* Interleukin-1 receptor blockade improves survival and hemodynamic performance in *Escherichia coli* septic shock, but fails to alter host responses to sublethal endotoxemia. *J Clin Invest* 1992; **89**: 1551–1557.
14. Strike PW. *Statistical Methods in Laboratory Medicine*. London: Butterworth-Heinemann, 1991; 108–128.
15. Casey LC, Balk RA, Bone RC. Plasma cytokine and endotoxin levels correlate with survival in patients with the sepsis syndrome. *Ann Intern Med* 119; **8**: 771–778.
16. Chang DM. The role of cytokines in heat stroke. *Immunol Invest* 1993; **22**: 553–561.
17. Heinrich PC, Castell JV, Andus T. Interleukin-6 and the acute phase response. *Biochem J* 1990; **265**: 621–636.
18. Bouchama A, Parhar RS, El-Yazigi A, Sheth K, Al-Sedairy S. Endotoxemia and release of tumor necrosis factor and interleukin 1 alpha in acute heatstroke. *J Appl Physiol* 1991; **70**: 2640–2644.
19. Wildeboor J, Camp J. Heat stress: its effect and control. *AAOHN J* 1993; **41**: 268–274.

**Received 5 December 1996; accepted 13 January 1997**
